# The New York City Department of Health and Mental Hygiene School Vision Program: A description of program expansion

**DOI:** 10.1371/journal.pone.0261299

**Published:** 2022-01-19

**Authors:** Sophia Day, Emanuela Acquafredda, Jill Humphrey, Martha Johnson, Maria Fitzpatrick, Jasmina Spasojevic, Kevin Konty

**Affiliations:** 1 NYC Office of School Health, New York, New York, United States of America; 2 Independent Scholar, New York, New York, United States of America; 3 New York City Department of Health and Mental Hygiene, New York, New York, United States of America; 4 Cornell University, Ithaca, New York, United States of America; Drexel University, UNITED STATES

## Abstract

**Objective:**

This study describes how the School Vision Program (SVP) operates in NYC Public Schools, and how it has expanded to provide screening, follow-up, eye exams, and even glasses to more students in recent years.

**Methods:**

Using administrative data from the SVP, we analyze a population sample of all public-school students with non-missing demographic variables in grades Pre-K through 12, focusing on the most recent year of data, 2018–19. We tabulate rates of screening and other results across students by grade and student characteristics, highlighting the expansion of SVP in community schools beginning in 2015–16.

**Results:**

The SVP screens about 87% of students in Pre-K through 1^st^ Grade each school year. Of the 22% of screened students who failed the screening in 2018–19, 69% received follow-up efforts, and 39% completed eye exams. Among students with completed eye exams, 13% of students in Pre-K through 1^st^ grade were diagnosed with amblyopia, and 70% needed glasses. Less advantaged students in terms of race, ethnicity, and socioeconomic status were less likely to pass vision screenings and less likely to receive eye exams after failing the screening. The SVP’s expansion to all grades in community schools and its provision of eye exams and glasses increased the rate of eye exams to 90% of students with a failed vision screening and distributed glasses to over 22,000 students in grades Pre-K to 12 in 2018–19.

**Conclusion:**

The expansion of SVP services in community schools suggests large potential benefits from school districts connecting students who fail vision screenings directly to eye doctors. Otherwise, low rates of follow-up eye exams in younger grades can lead to unidentified and unmet need for vision services in older grades, especially among disadvantaged students.

## Background

### School-based vision services in the United States

Delivering high-quality health programs in a school-based setting provides a promising approach to eliminating health disparities, as services offered during school hours and on school grounds yield exceptional reach [1]. The critical link between the health status of children and their educational success has propelled the implementation of diverse health programming in schools. The Office of School Health (OSH), a joint bureau of the New York City Department of Health and Mental Hygiene (DOHMH) and Department of Education (DOE), provides a broad range of health services to students in New York City (NYC) schools. Specific programs, like the School Vision Program (SVP), seek to identify students throughout the NYC school system who are at high risk for vision deficiencies that may be hindering their academic achievement.

Vision deficiencies, including refractive error and other vision disorders, are common among school-age children, yet most are treatable if caught early. A report from the National Academies of Sciences, Engineering, and Medicine (NASEM) details the prevalence and treatability of different types of vision problems among children in the United States [2]. According to that report, refractive errors, which include conditions such as myopia, hyperopia, and astigmatism, are present in at least 20% of children. These conditions can be corrected by adhering to prescribed eyeglass use. Amblyopia, commonly referred to as “lazy-eye,” affects an estimated 1–3% of young children and can lead to blindness in one or both eyes if gone undetected in early childhood. Strabismus, a condition that interferes with an individual’s depth perception and often leads to amblyopia, affects 2–4% of young children. Though not as easily corrected as refractive error, the early detection of amblyopia and strabismus provides the best opportunity for effective treatment [2].

Once detected, it is important for children with vision deficiencies to receive follow-up care after a failed screening and adhere to treatment. Visual impairments noticed but unattended to during early childhood may lead to worsened visual acuity as well as academic underperformance. Unclear vision is a significant barrier to literacy, and students who do not reach the desired reading levels by the end of 1^st^ grade miss the first benchmark of academic achievement [3]. Students who may have trouble reading or seeing blackboards may abstain from participating in class discussions and show overall decreased motivation in the classroom [3,4]. In addition, difficulty seeing may impair children’s physical health if it makes physical activity more difficult.

There are clear disparities in vision treatment by race and socio-economic status as well as some documented differences in underlying vision health. A 2012 analysis of trends in racial/ethnic disparities of child health in the U.S. showed that Black children had significantly higher reports of vision problems compared to White children [5]. Summarizing studies that have measured refractive error among young children by race and ethnicity, Welp and colleagues report somewhat higher rates of astigmatism among Hispanic children relative to non-Hispanic children, but higher rates of hyperopia among non-Hispanic White children relative to non-Hispanic Black and Hispanic children [1]. Among older children, ages 12–19, Qiu and colleagues find significant disparities in vision treatment by both race/ethnicity and socioeconomic status (SES), with Hispanic and non-Hispanic Black teenagers and those of lower SES less likely to have their refractive errors adequately corrected [6]. Patterns in large U.S. cities like Philadelphia and Los Angeles similarly show that a child’s race/ethnicity and SES are correlated with accessing eye care when needed [7,8].

School-based vision programs that serve diverse populations are well-positioned to reduce disparities in vision acuity and in access to vision care. However, early studies of the effectiveness of traditional school-based vision programs found that many students who fail vision screenings do not ultimately receive treatment [9]. This is an issue in school-based vision programs that provide screenings only and recommend families to seek vision care. Shakarchi and Collins review a dozen studies of school-based vision programs that provide screening as well as eye-exams and glasses prescriptions [9]. They refer to these as “school-based eye care programs” to contrast them with traditional screening programs (p. 860). Though varying widely in design and number of students served, these programs achieved high rates of eye-exams among those failing the initial vision screening. These programs include the Baltimore Vision Project, Philadelphia Eagle Eye Mobile, UCLA Preschool Vision Program, and Toledo Public Schools Eye Care Program, among others [9]. Our analysis of the NYC School Vision Program contributes to the literature that describes and evaluates such programs.

We provide a descriptive analysis of the expansive School Vision Program operating in NYC public schools. In addition to providing a detailed picture of screening, follow-up efforts, eye exams, and abnormal diagnoses in school year 2018–19, we describe how the program’s reach has expanded over time since 2010–11. In particular, we report rates of screening failure, follow-up exams, and diagnoses after the SVP expanded in a subset of high-need schools to cover older students and to provide eye exams directly. The results of this analysis have lessons for other school districts seeking to expand the reach and effectiveness of their vision screening programs.

### The NYC Office of School Health School Vision Program

The School Vision Program (SVP), housed in the Office of School Health, was formed after state and city-level mandates required that, at a minimum, all students be screened for vision deficiencies. New York State vision screening guidelines mandate that in-school screenings be conducted for all new entrants, within six months of enrollment, Kindergarten, 1^st^, 3^rd^, and 5^th^ grade students. NYC policies indicate that the DOHMH be responsible for conducting all Pre-K (PK), Kindergarten (K), and 1^st^ grade screenings, and the SVP implements these DOHMH vision screenings [10].

In recent years NYC has expanded the SVP in community schools by screening students in all grades PK-12 and by providing vision care directly, including eye exams and free glasses. Community schools are a subset of 250-plus high-need schools that offer additional social and health services to students and their guardians. Located throughout the city and including elementary, middle, and high schools, these schools were selected for the program based on low attendance, high drop-out rates, and lower academic performance. Expansion of the SVP in community schools began in the 2015–16 academic year. Screenings in community schools cover grades 2–12 in additional to the regular screening for grades PK-1. Mayoral funding and public-private partnerships additionally enable all students in these settings to receive eye exams and glasses, if needed. While students in community schools are a somewhat unrepresentative subset of NYC students due to how schools were selected, the results that we present here are suggestive of the benefits of expanding the SVP to other schools.

## Data and methods

### Sample construction

We use administrative data from the SVP linked to student demographic data from the DOE covering students who attended public schools in grades PK-12, from 2010–11 to 2018–19. Variables related to vision screening and follow-up come from data reported by SVP staff, and variables related to eye exams and abnormal diagnoses were entered by SVP staff from forms filled out by the optometrist who performed the student’s eye exam.

Among about 2.5 million students who appear as screened through SVP, 2.3% do not appear in DOE demographic student files, possibly due to leaving the public-school system before the end of the school year and are not included in our sample. Out of about 10.2 million students, screened and unscreened, in the demographic student files, 2.2% are either missing grade information or are not in grades PK-12. An additional 0.9% are missing at least one of gender, race/ethnicity, or free or reduced-price lunch status variables, leaving a sample of 9,901,434 students across all years and 1,107,685 students in 2018–19.

### Study design

This paper aims to use the rich administrative data collected through the SVP over the last several years to describe in detail how the program operates and how it has expanded to reach more students with more vision care services over time. The size of the SVP population and level of detailed data collected and entered by SVP staff allows us to provide a fuller picture of a school vision program (and the prevalence of vision problems it uncovers) than has previously been available. Rather than test a hypothesis of the effect of SVP on student outcomes or test for differences in vision by student characteristics, we focus on tabulating several key measures that capture the direct outputs of the SVP: the rate of vision screening among enrolled students, the rate of screening failure among screened students, the rate of follow-up among students that fail vision screenings, the rate of confirmed eye exams among students that fail vision screenings, and the rates of various abnormal diagnoses among students with confirmed eye exams.

Because the main population served by the SVP are students in grades PK-1, our analysis focuses on this population. However, by showing the same key measures of interest for older students in community schools in 2015–16 and later, we show just how many students fall through the cracks, such that their vision problems persist unresolved or develop at an older age. Furthermore, we show the difference between the rate of confirmed eye exams among students that fail vision screenings when those eye exams are provided directly through the SVP rather than placing that burden on students’ families.

### Description of population

The NYC public school system is the largest in the U.S. and has a diverse student body in race/ethnicity and SES. In the 2018–19 academic year, 1,107,685 total students were enrolled in the NYC public schools in grades PK-12 (Table 1). Of the total enrolled population, 51.4% of students were male, and approximately 15.3% were non-Hispanic White (White), 25.7% non-Hispanic Black (Black), 41.1% Hispanic, 16.5% Asian or Pacific Islander (Asian), and 1.5% multiracial or missing race/ethnicity information (other). About 74.1% were identified as individually eligible for free or reduced-price lunch or Human Resources Administration (HRA) benefits (e.g., Supplemental Nutrition Assistance or Temporary Assistance for Needy Families), an indicator used to characterize children living in low-income households. Beginning in school year 2017–18, all students received free school meals throughout NYC schools. However, the Department of Education continued to identify students individually eligible for purposes of reimbursement from the National School Lunch Program. (Note that the free or reduced-price lunch variable is an imperfect measure of economic disadvantage, because not all income-eligible families provide this information to schools, and neither do all income-eligible families participate in SNAP or TANF.)

**Table 1:** Summary of demographic characteristics of students enrolled in NYC public schools and students screened by the School Vision Program, 2018–19.

	All Students		Screened Students		
Characteristic	Number	Percent of Total	Number	Percent of Total	Percent Screened
Grade					
Pre-K	71,979	6.5	56,258	21.2	78.2
Kindergarten	77,245	7.0	66,827	25.2	86.5
1st Grade	80,610	7.3	69,999	26.4	86.8
2nd-5th Grades	323,718	29.2	31,250	11.8	9.7
6-8th Grades	236,683	21.4	20,505	7.7	8.7
9-12th Grades	317,450	28.7	20,673	7.8	6.5
Gender					
Male	569,128	51.4	136,477	51.4	24.0
Female	538,557	48.6	129,035	48.6	24.0
Race					
Hispanic	454,910	41.1	114,696	43.2	25.2
Non-Hispanic Black	284,335	25.7	62,603	23.6	22.0
Asian/Pacific Islander	183,060	16.5	42,785	16.1	23.4
Non-Hispanic White	169,299	15.3	42,141	15.9	24.9
Multiracial or Missing	16,081	1.5	3,287	1.2	20.4
Borough of School District					
Manhattan	138,051	12.5	31,272	11.8	22.7
Bronx	196,690	17.8	52,800	19.9	26.8
Brooklyn	282,147	25.5	73,275	27.6	26.0
Queens	282,595	25.5	67,875	25.6	24.0
Staten Island	61,300	5.5	13,181	5.0	21.5
Non-Geographic Districts	146,902	13.3	27,109	10.2	18.5
Free or Reduced-Price Lunch					
Eligible	820,975	74.1	195,487	73.6	23.8
Not Eligible	286,710	25.9	70,025	26.4	24.4
Total	1,107,685	100	265,512	100	24.0

Notes: These data come from a merge between DOE’s student demographic data file and the SVP’s student program data file. Screened students are a subset of enrolled students; the rightmost column is equal to the number screened divided by the number enrolled. Non-Geographic Districts include alternative schools and schools serving students with disabilities.

Among those enrolled in 2018–19, about 24.0% received in-school vision screening. Screening rates were between 78.2% and 86.8% among students in grades PK-1; students DOHMH is responsible for screening. Those not screened in these younger grades were absent from school or could not be tested because of other outstanding medical issues. Those screened in older grades attend community schools. Demographic characteristics of the screened population closely match those among the broader enrolled population.

## School Vision Program for Pre-K, Kindergarten, and 1st grade students

### Overview of program procedures

The school vision program operates through several teams that collaborate to provide vision screenings, follow up with families, perform eye exams through the optometry program, and enter data into student health records. Twelve K-1 teams are assigned geographically, led by a Team Leader and Public Health Advisor, and staffed by three Public Health Assistants. An optometrist provides technical assistance and consultation to the teams and OSH school-based staff. The Team Leader plans the logistics of the screening with the respective principals and is responsible for all statistical data, while the Public Health Advisor coordinates with school nurses. Screeners measure distance and near vision, fusion, and color vision. The teams use one of three tests (Snellen, tumbling E, or Lea Symbol chart) for distance vision. Near vision is tested with a Rosenbaum pocket chart and a +2.50 flipper lens. Color vision is tested with an Ishihara unlettered version book, and fusion with the Lang stereo test. The teams are responsible for entering all screening data into ASHR using laptops provided by the program.

The PK screening staff, consisting of Public Health Advisors, use the Welch-Allyn Sure Sight and SPOT auto-refractors to screen children in the DOE Universal Pre-K Program. The Lea Symbol Chart may also be used if a K/1^st^ grade screening team member screens a PK student. The OSH has a policy agreement with the DOE to provide this screening service, both in public schools and in community-based organizations. PK screeners schedule and screen their schools and enter their screening results into the Automated Student Health Record (ASHR).

Children who fail vision screening in PK, K, or 1^st^ grade, or who cannot be tested, are sent home with 1) a letter advising the parent to take the child to an eye doctor, 2) an eye report and recommendation form (E12s) for the eye doctor to fill out, 3) a list of public facilities that will enroll children without insurance, and 4) a pre-addressed, postage paid envelope in which the parent can return the completed eye form to the OSH.

Follow-up begins immediately after screening, with calls alerting parents to the letter and E12s form that will be in their children’s backpack. The follow-up unit, which consists of one supervisor and five Public Health Advisors, receive priority lists from field staff to contact parents of children in grades K-1 based on their vision screening results. In grades K-1, children receive priority for follow-up when their vision screening score is 20/70 or worse, or when their results suggest a risk for amblyopia due to a difference of two lines or more between right and left eyes in distance vision. All PK students who fail the screening should also receive follow-up.

Subsequent calls are made every two weeks to encourage and assist the parent in finding pediatric eye care. If the family cannot be reached by phone, the unit sends a letter. Some families require only one phone call to take their child to the eye doctor; others require innumerable intervention attempts. For families seeking assistance with eye care providers, the follow-up unit refers children to select ophthalmologists. The unit assists families in need of health insurance and visits schools if necessary to contact hard-to-reach families. The goal is to get every child with poor vision to an eye doctor.

Students with confirmed eye exams have returned referral E12s forms with exam results as filled out by their eye doctor. The data entry unit, consisting of one supervisor and four staff members, enters the data in these returned forms into ASHR. The staff of the data entry unit is also responsible for collecting any missing information on the E12s forms by contacting the doctor’s office or contacting parents. After receiving all the missing information, the updated data are entered into ASHR and a copy of the E12s form is mailed to the school nurse for the child’s file.

### Screening rates

In school year 2018–19, about 78% of PK students received the in-school vision screening described above, while about 87% of students in grades K-1 were screened (Table 2). Attendance is often lower in PK than later grades [11]; lower attendance on vision screening days may partially explain a lower rate of screening in PK. Rates of screening among grades PK-1 have changed little over time since 2010–11, ranging from about 87 to 88% each year.

**Table 2:** Rates of screening, failed screening, follow-up, and confirmed exams in Pre-K to 1^st^ grade, 2018–19.

	Enrolled	Screened	Failed Screening	Followed-up	Exam Confirmed
	Number	(% of Enrolled)	(% of Screened)	(% of Failed Screenings)	
Pre-K	71,979	78.2	22.3	97.3	42.2
Kindergarten	77,245	86.5	23.3	57.0	37.2
1st Grade	80,610	86.8	21.0	57.9	37.7
Total	229,834	84.0	22.2	69.1	38.8

Notes: These data come from a merge between DOE’s student demographic data file and the SVP’s student program data file. While some students who passed the vision screening obtain eye exams, the “Exam Confirmed” column is calculated using only those who also failed the vision screening.

### Screening failure and follow-up rates

Among the 193,084 students in grades PK-1 screened in 2018–19, about 22.2% failed the screening. This percentage varied little by grade, with between 21.0% and 23.3% failing the screening in each of these three grades (Table 2). Rates of vision screening failure have changed little over time since 2010–11 when 19.6% of screened students in grades PK-1 failed the screening. What has changed over this time period are rates of follow-up in response to failed screenings.

In 2018–19, among 42,827 students in grades PK-1 that failed their vision screenings, about 69.1% received follow-up efforts from staff (Table 2). This number averages a high rate of follow-up among PK students, 97.3%, and rates of 57.0% and 57.9% for students in Kindergarten and 1^st^ grade, respectively. These percentages translate into nearly 30,000 students in these grades that received follow-up communication efforts following vision screening failure. Rates of follow-up for students who failed vision screenings in grades PK-1 have increased over time, from 42.7% in 2010–11 to nearly 70% in the latest year of data.

### Rates of confirmed eye exams

Despite follow-up efforts, not all students who fail their vision screening will complete an eye exam and return the E12s form with their exam results. Among those students who failed the vision screening in 2018–19, 38.8% were confirmed to have had eye exams because they returned completed E12s forms (Table 2). Rates were slightly higher for students in PK than for students in grades K-1 (42% versus 37%), but not as much higher as the rate of follow-up for PK would suggest (97% versus 57%). Similarly, while rates of confirmed exams have increased slightly since 2010–11 (from 37.7% in 2010–11), they have not increased in proportion to the increase in follow-up rates.

### Rates of abnormal diagnoses

Among those students confirmed to have received eye exams (E12s forms returned), about 90.6% received diagnoses of vision problems from their optometrists (Table 3). Optometrists can report up to three diagnoses for each student. In 2018–19, about 13.0% of students with confirmed eye exams were diagnosed with amblyopia, 0.3% with strabismus, 31.1% with hyperopia, 23.8% with myopia, and 66.6% with astigmatism (note that these are not mutually exclusive categories). (We identify students as having: (1) amblyopia if any of their diagnoses are “Amblyopia”, “Refractive/ametropic amblyopia”, “Strabismic amblyopia”, “Deprivational amblyopia”, “Organic amblyopia”, or “Meridional amblyopia”; (2) strabismus if any of their diagnoses are “Strabismus” or “Strabismic amblyopia”; (3) hyperopia if any of their diagnoses are “Hyperopia/hypermetropia” or “Hyperopic astigmatism”; (4) myopia if any of their diagnoses are “Myopia” or “Myopic astigmatism”; and (5) astigmatism if any of their diagnoses are “Astigmatism”, “Hyperopic astigmatism”, or “Myopic astigmatism”.) About 69.9% of students with confirmed eye exams were determined by optometrists to need glasses, which translates to about 5.2% of students in grades PK-1 determined to need glasses through the School Vision Program’s screenings.

**Table 3:** Rates of abnormal diagnoses among screened students with eye exams confirmed in Pre-K through 1^st^ grade, 2018–19.

Grade	Exam Confirmed	Any Abnormal Diagnosis	Amb.	Strab.	Hyp.	Myop.	Astigm.	Needs Glasses
	Number	(% among Students with Exam Confirmed)						
Pre-K	5,355	91.8	14	0.2	33.0	16.9	75.4	64.6
Kindergarten	6,076	89.2	13.6	0.4	31.2	22.8	63.0	67.7
1st Grade	5,785	91.1	11.6	0.4	29.3	31.1	62.2	77.0
Total	17,216	90.6	13.0	0.3	31.1	23.8	66.6	69.9

Notes: This table includes all students with confirmed eye exams, including those that passed the vision screening. The diagnoses are not mutually exclusive, and “Any Abnormal Diagnosis” encompasses more diagnoses than those listed in the table.

Rates of different vision problems varied somewhat by grade. Rates of myopia and strabismus increased with grade, while rates of amblyopia, hyperopia, and astigmatism decreased with grade. The rate of needing glasses increased with grade from 64.6% among Pre-K students to 77.0% among 1^st^ graders who received eye exams. It is important to note that these patterns by grade reflect differences in which students obtain eye exams as well as differences in the underlying prevalence of vision conditions by grade. For example, the prevalence of amblyopia may decline with grade in this data partially because students with signs of amblyopia already received the diagnosis after an earlier screening.

Rates of abnormal diagnoses have also changed somewhat over time. Rates of any abnormal diagnosis have remained close to 90% over the years, and rates of needing glasses have varied between 70 and 75%. Rates of hyperopia have increased slightly, rates of amblyopia have decreased slightly, and rates of astigmatism have increased from about 58% to about 68% over this time period among students with confirmed exams.

### Variation by student and school characteristics

In this section, we examine how rates of screening, screening results, follow-up, exams, and exam results among students in grades PK-1 varied by student and school characteristics in school year 2018–19. We show in Table 4 how these rates vary by students’ gender, race/ethnicity, and SES, as well as absence rate and whether students attended a community school or not.

**Table 4:** Rates of screening, failed screenings, follow-up, and confirmed exams by student and school characteristics, among students in Pre-K through 1^st^ grade, 2018–19.

	Enrolled	Screened	Failed Screening	Followed-up	Exam Confirmed
Characteristic	Number	(% of Enrolled)	(% of Screened)	(% of Failed Screenings)	
Gender					
Female	113,095	84.2	21.5	70.3	38.9
Male	116,739	83.8	22.8	68.1	38.7
Race					
Hispanic	92,360	81.6	27.3	69.7	37.2
Non-Hispanic Black	53,681	79.7	22.9	66.2	28.8
Asian/Pacific Islander	39,653	89.2	19.3	72.9	53.3
Non-Hispanic White	41,204	90.1	13.6	67.6	45.6
Multiracial or Missing	2,936	82.4	24.0	66.1	38.7
Free or Reduced-Price Lunch					
Eligible	161,238	82.5	25.0	67.8	37.2
Not Eligible	68,596	87.6	16.0	73.5	44.4
Absence					
Chronically Absent	51,183	78.7	25.7	62.4	38.9
Not Chronically Absent	181,010	88.7	19.7	67.9	46.0
School Type					
Community School	15,338	92.5	33.3	N/A	85.6
Regular School	214,496	83.4	21.3	68.4	33.0
Total	229,834	84.0	22.2	69.1	38.8

Note: These data come from a merge between DOE’s student demographic data file and the SVP’s student program data file. While some students who passed the vision screening obtain eye exams, the “Exam Confirmed” column is calculated using only those who also failed the vision screening.

Rates of follow-up in community schools are not applicable, because screening and exams are both provided by the SVP. Rates by absence use data from 2017–18 due to data availability. Chronically Absent is defined as missing 18 or more days of school (10% of school days). Regular Schools are defined as all schools that are not Community Schools.

Rates of screening, follow-up, and exams vary by how often students are absent from school and by whether students attend a community school, as these can directly determine access to services. Students who are absent more often are more likely to miss the day of vision screening, and students who attend community schools have access to free eye exams, as discussed in the next section. Chronically absent (missed 18 or more school days) students were ten percentage points less likely to be screened for vision than those who were not chronically absent (78.7% versus 88.7%). Students who attended community schools were nearly ten percentage points more likely to be screened (92.5% versus 83.4%) and significantly more likely to receive eye exams (85.6% versus 33.0%).

While SVP screenings are provided to students of all backgrounds, disparities in screening by absenteeism carry over to disparities by race/ethnicity and SES. White and Asian students were screened at a rate of about 90%, while Hispanic, Black, and other students were screened at closer to 80% rate. Students not eligible for free or reduced-price lunch were five percentage points more likely to receive screenings.

Disparities in vision screening failure are more significant. Conditional on being screened, Hispanic students failed the vision screening at twice the rate of White students, and students eligible for free or reduced-price lunch failed at a rate about 50% higher than other students. Since screening is done with glasses if a child already has them, this may be because these students are less likely to have received screening and treatment for vision problems before school entry than their counterparts.

While follow-up efforts were relatively similar across racial/ethnic groups, White and Asian students were still more likely to have confirmed eye exams than Hispanic and Black students conditional on having failed the vision screening (see Table 4). Similarly, students who were ineligible for free or reduced-price lunches were more likely to get eye exams after failing the screening.

Conditional on having confirmed eye exam results, which varies by student characteristics as described above, the rates of different abnormal vision diagnosis also vary by student characteristics. As shown in Table 5, students ineligible for free or reduced-price lunch were less likely to receive most of these abnormal diagnoses and less likely to need glasses. The exceptions were amblyopia and hyperopia, with which students not eligible for free or reduced-price lunch were slightly more likely to be diagnosed. Differences by race/ethnicity were larger in magnitude and more complex. For example, Black students with confirmed exams were most likely to need glasses (75.9%) and most likely to be diagnosed with myopia (29.1%), but least likely to be diagnosed with amblyopia (10.7%). White students were least likely to need glasses (59.5%), but most likely to be diagnosed with hyperopia (38.9%) and amblyopia (15.4%). Hispanic students were most likely to be diagnosed with astigmatism (72.5%). These diagnoses rates are only measured among students with completed eye exams, making it difficult to interpret the differences in diagnoses as differences in underlying prevalence. However, the higher rate of astigmatism among Hispanic students and the higher rate of hyperopia among non-Hispanic White students are consistent with other studies of refractive error among children by race and ethnicity [2].

**Table 5:** Rates of abnormal diagnoses by student characteristics, among students in Pre-K through 1^st^ grade, 2018–19.

	Exam Confirmed	Any Abnormal Diagnosis	Amb.	Strab.	Hyp.	Myop.	Astigm.	Needs Glasses
Characteristic	Number	(% among Students with Exam Confirmed)						
Gender								
Female	8,242	90.8	13.7	0.3	31.4	23.5	67.3	71.1
Male	8,974	90.5	12.4	0.4	30.8	24.1	66.0	68.7
Race								
Hispanic	7,865	91.3	13.2	0.4	31.8	21.4	72.5	73.4
Non-Hispanic Black	2,875	91.8	10.7	0.4	28.8	29.1	69.1	75.9
Asian/Pacific Islander	3,852	90.7	13.1	0.2	27.2	28.4	62.2	64.3
Non-Hispanic White	2,390	87.0	15.4	0.4	38.9	16.9	51.6	59.5
Multiracial or Missing	234	91.0	11.1	0.0	21.8	32.5	64.5	71.8
Free or Reduced-Price Lunch								
Eligible	12,778	91.1	12.5	0.4	30.9	24.3	68.6	71.8
Not Eligible	4,438	89.3	14.7	0.2	31.8	22.2	61.0	64.3
Total	17,216	90.6	13.0	0.3	31.1	23.8	66.6	69.9

Notes: This table includes all students with confirmed eye exams, including those that passed the vision screening. The diagnoses are not mutually exclusive, and “Any Abnormal Diagnosis” encompasses more diagnoses than those listed in the table.

## Optometry programs for at-risk students

In addition to the vision screening and follow-up efforts described above, OSH also provides free eye exams and glasses to certain at-risk students. In its longest-running optometry program, OSH has provided eye exams and glasses to students in grades 1–3 who are at the highest risk for amblyopia since 2010–11. In a new program begun in 2016–17, OSH provides these same services to students of all grades whose families live in temporary housing.

The optometry program for students at risk of amblyopia operates in a subset of NYC public schools, chosen based on the number of students who failed vision screenings but did not see an eye doctor in the previous school year. Optometrists hired as medical consultants for the DOHMH conduct eye exams for those students at risk for amblyopia who do not return medical documentation from an eye care provider through this program. If the optometrist determines that a student requires eyeglasses, optical vendors under contract to the DOE provide the eyeglasses. In the most recent year for which data are available, 2017–18, this program operated in 250 schools, providing over 4,500 students with eye exams and providing 3,932 pairs of glasses.

OSH provides almost as many free pairs of glasses through a newer program begun in school year 2016–17 for students in temporary housing. Like the program for students at risk of amblyopia, the program for students in temporary housing operates in a subset of schools identified by the DOE as having the largest number of students in temporary housing. In 2017–18, this program provided about 3,500 students across 49 schools with free eye exams and glasses. While not as large as the community schools expansion discussed in the next section, these optometry programs deliver targeted services to subgroups of hard-to-reach students.

## Community school expansion

### SVP in community schools

School Vision Program services have expanded to include all grades in NYC’s community schools. These schools are identified based on academic vulnerability but represent an opportunity to provide expanded services to more students who may also be at risk for poor health. Expansion of the SVP began in the 2015–16 academic year, with an additional 32,874 students in grades 2–12 receiving vision services through programming in community schools. Since that time, the number of community schools has increased, and with it, the number of students receiving vision screening through this expansion, to 72,428 students in grades 2–12 in school year 2018–19.

Most community schools are classified as Attendance Improvement and Drop-Out Prevention (AIDP) Schools or Renewal Schools. AIDP schools are 45 grant-funded schools that follow the classic, national community school model by partnering with community organizations to offer various social and behavioral services for both students and guardians. Renewal Schools selected for the mayoral initiative met several criteria for being low-performing schools academically.

Mayoral funding and public-private partnerships with the nonprofit organization Helen Keller International (HKI) and the optical company Warby Parker have enabled all students in these settings to receive free eye exams and glasses if needed. By expanding the reach of SVP, children in community schools who may have been missed in early age screenings or require further treatment can be attended to throughout their academic years. This may also include students who have been unable to adhere to prescribed treatment if they were screened in previous years.

### Screening and eye exam procedures and rates in community schools

Initial vision screening in community schools follow the model of PK to 1^st^ grade programming. Then, unlike regular PK-1 programming, optometrists from the OSH optometry program and HKI provide eye exams to most students in community schools who fail the initial screening, often on the same day. Other students may be referred out for additional services. For those students whose eye exams show that they need glasses, prescriptions are sent to Warby Parker, which delivers free glasses to students via OSH and HKI staff. Throughout this process, staff also update ASHR with information about students’ eye exam results and receipt of glasses.

In school year 2018–19, 86,530 students in grades PK-12 were enrolled in community schools (Table 6). Rates of screening were between 90 and 95% for students in grades PK-8 and about 81% for students in high school grades. Rates of failing the vision screening remained high in older grades, with rates of failure among screened students in high school comparable to failure rates among screened students in Kindergarten (33.3% and 32.7%, respectively).

**Table 6:** Rates of screenings, failed screenings, follow-up, and confirmed exams in community schools by grade, 2018–19.

Grade	Enrolled	Screened	Failed Screening	Exam Confirmed
	Number	(% of Enrolled)	(% of Screened)	(% of Failed Screenings)
Pre-K	2,894	91.3	23.5	84.2
Kindergarten	5,946	92.4	32.7	84.8
1st Grade	6,498	93.1	38.1	86.6
2nd-5th Grades	26,379	94.5	41.1	90.2
6-8th Grades	20,163	94.9	39.3	89.2
9-12th Grades	24,650	81.2	33.3	91.9
Total	86,530	90.4	37.3	89.6

Note: These data come from a merge between DOE’s student demographic data file and the SVP’s student program data file. While some students who passed the vision screening obtain eye exams, the “Exam Confirmed” column is calculated using only those who also failed the vision screening. Rates of follow-up in community schools are not applicable, because screening and exams are both provided to these students by the SVP.

Because the SVP directly provides eye exams to students in community schools, follow-up efforts are not needed to remind families to set up eye appointments, and the rates of confirmed eye exams are high. Among these students who failed vision screenings in community schools, about 90% received confirmed eye exams across all grades. Rates of eye exams were even higher among 2^nd^ to 12^th^ graders than among grades PK-1.

### Rates of abnormal diagnoses and receiving glasses in community schools

Among those students in community schools that received eye exams in 2018–19, 94.5% had some abnormal diagnosis, and 86.3% were determined to need glasses (Table 7). Most of these students were diagnosed with astigmatism (67.6%), about half with myopia (50.6%), 22.8% with hyperopia, 4.1% with amblyopia, and 0.2% with strabismus. Abnormal diagnoses varied significantly by grade. About 94.2% of students in high school were determined to need glasses compared to 65.0% of students in Pre-K. Older students were also much more likely to be diagnosed with myopia (71.2% of high school students versus 14.2% Pre-K students). Rates of the other diagnoses shown in Table 7 declined with grade. To put this in perspective, these numbers suggest that 25% of high schoolers in these schools need glasses but do not have or wear them without the vision program. More generally, 30% of children in these schools have some form of vision problem that needs addressing.

**Table 7:** Rates of abnormal diagnoses among screened students with confirmed exams in community schools by grade, 2018–19.

Grade	Exam Confirmed	Any Abnormal Diagnosis	Amb.	Strab.	Hyp.	Myop.	Astigm.	Needs Glasses
	Number	(% among Students with Exam Confirmed)						
Pre-K	535	88.6	7.1	0.4	37.8	14.2	70.5	65.0
Kindergarten	1,543	90.0	7.6	0.3	35.3	15.7	74.7	74.1
1st Grade	2,004	90.0	5.1	0.3	33.8	22.1	73.6	76.2
2nd-5th Grades	9,270	93.2	4.6	0.2	27.4	41.6	67.2	83.7
6-8th Grades	6,723	96.0	2.9	0.1	17.1	63.6	64.2	90.2
9-12th Grades	6,135	98.1	3.3	0.2	14.0	71.2	68.0	94.2
Total	26,210	94.5	4.1	0.2	22.8	50.6	67.6	86.3

Notes: This table includes all community-schools students with confirmed eye exams, including those that passed the vision screening. The diagnoses are not mutually exclusive, and “Any Abnormal Diagnosis” encompasses more diagnoses than those listed in the table.

Through the partnership with HKI and Warby Parker, students in community schools not only receive eye exams when necessary, but also receive glasses if eye exams confirm a need for glasses. After the first year of the partnership with Warby Parker, the rate of students receiving glasses increased from 70.0% in 2015–16 to very close to 100% in the next three years. Over half of students that received glasses in 2018–19 were in grades 6–12, which highlights the level of unmet need in vision treatment that the SVP has identified and begun to address.

## Limitations

There are several limitations to the analysis presented here. First, our sample doesn’t capture the full population served by the SVP because we exclude students outside the public school system. Second, our relatively simple measures of screening, follow-up, and exams do not capture variation within these measures. For example, some students are screened multiple times in the same school year, and students are screened on various aspects of their vision. Thus, our measure of screening failure doesn’t capture the difference between failing one element of the vision screening versus failing all elements. Because we do not capture those differences, we also do not report here to what extent those students who ultimately receive eye exams are the students who failed the vision screening to a lesser or greater extent. Finally, our measure of the rate of confirmed eye exams is lower bound on the rate of completed eye exams, because it is based on returned E12s forms, or on the success of SVP staff in tracking down students’ eye exam results. Some students who do get eye exams may just fail to return the paperwork.

## Discussion

As a joint program of the DOHMH and the DOE, the Office of School Health’s School Vision Program provides universal vision screening to students in grades PK to 1. As documented here, its efforts have expanded over time to provide more direct vision treatment in some schools. The follow-up unit and optometry program each do their part to increase access to treatment, targeting students with the worst vision or at the highest risk for amblyopia. Expanded programming in community schools provides eye exams on-site as well as free glasses through partnerships with HKI and Warby Parker. Despite community schools being chosen based on academics rather than vision screening results, universal screenings there have identified a high need for vision care, even among students in older grades.

The higher rates of eye exams in community schools, coupled with the finding that at least five percent of students without previously diagnosed vision issues are found to have serious issues requiring treatment, suggests that the SVP’s expanded programming may be worth emulating across all NYC schools and in other school districts with similar populations. Care should be taken to ensure that students receive follow-up exams within the system since, without these, many students would go without treatment for their diagnosis.

Providing treatment within the school setting is also a way to address disparities in vision and disparities in access to treatment by race/ethnicity and socioeconomic status. In recent years the DOHMH has focused more explicitly on health equity, noting how a history of systemic racial discrimination in the city has led to health disparities between communities of color and White communities [12,13]). We see this inequity reflected in worse vision outcomes and worse access to vision care among Black and Hispanic students in SVP program data. Among students screened for vision in PK-1, we find a higher rate of vision screening failure among Black and Hispanic students than White and Asian students. Among those who fail the vision screening and need follow-up exams, we find that these same groups are less likely to have confirmed eye exams. By providing eye exams and glasses directly, the SVP ensures that all students, regardless of race/ethnicity, have immediate access to vision care. However, even following this strategy, the SVP and similar programs must be careful not to miss students absent on the day of vision screening.

This work also reflects on the importance of high-quality data to understand the full scope and reach of school programming. This study was possible because of substantial careful effort to collect and catalog data on the SVP from initial screening to follow-up to treatment on all students. This coverage of the entire student population allows for identification in differential patterns of screening, diagnosis, and treatment not possible in other environments without such data. It also enhances understanding of program effectiveness, for example, by indicating the confirmed exam rates are much higher in community school settings where those exams are offered as part of the program.

While a link between students’ vision and academic achievement is plausible, there is an inherent need to look at holistic and comprehensive health models within schools. The services provided by the SVP extend beyond state and city mandates and offer opportunities for equitable access to optical clinical services and treatment. Barriers to effective care are often comprised of socioeconomic, educational, and health factors; thus, only a coordinated and simultaneous effort on all of these fronts can address the continuing unmet need and close the achievement gap [14].

Going forward, it will be important to understand the full costs and benefits of various school vision programs, including the iterations of the NYC program studied here. Doing so will require information on staffing and treatment costs and measures of the benefits to children and their families. Although fully measuring benefits to children and families may be difficult because they are likely to accrue across multiple dimensions, a better understanding of even how children’s academic and physical health outcomes improve with diagnosis and treatment may prove that these programs are worth the investment.
